# Curbing prescription opioid dependency

**DOI:** 10.2471/BLT.17.020517

**Published:** 2017-05-01

## Abstract

An epidemic of overdoses and deaths from opioids is fuelled by increased prescribing and sales in North America. Tatum Anderson reports.

He didn’t know it at the time, but Dr Andrew Kolodny had begun to witness the beginnings of a major drug addiction crisis in the United States of America (USA).

A public health expert on drug dependency at New York City’s health department in the early 2000s, Kolodny worked in poor neighbourhoods from South Bronx to central Brooklyn blighted by crack cocaine and heroin.

“It was while we were working on the illicit drugs problem that we noticed drug overdose deaths were increasing in middle-class communities in the New York City area,” he says.

Before 1995, the prescription of opioid painkillers in the USA was limited to people with pain from advanced cancer, severe injuries or after major surgery.

That restraint was founded on the fear that patients might become addicted and the bitter experience of two opioid epidemics: in the early 1900s when heroin was sold legally for various ailments and an epidemic of illegal heroin dependency in the 1960s during the Vietnam War.

Opioids are a class of drug used to reduce pain. They include heroin, as well as prescription pain relievers, such as oxycodone, hydrocodone, codeine, methadone and morphine.

In 1986, a study based on 38 patients suggested that these powerful prescription drugs could be used safely with minimal rates of addiction for chronic pain, and a few pain specialists began advocating for broader use of them in this way, says Kolodny, who is now the director of nongovernmental organization Physicians for Responsible Opioid Prescribing and a researcher at Brandeis University in Massachusetts.

This movement to increase opioid prescribing was catalysed in the 1990s by patient and professional groups that received funding from pharmaceutical companies, he says. 

Since then, the USA has been facing a growing epidemic of opioid dependency, including a rise in heroin use and record high levels of opioid overdose deaths. 

From 1999 to 2015, more than 183 000 people died from overdoses related to prescription opioids and in 2012, more than 250 million opioid prescriptions were written in the USA, according to the Centers for Disease Control and Prevention (CDC), more than enough for the entire population of 319 million people.

Some of these prescriptions were for palliative care or acute pain, but many more were for patients with chronic non-cancer pain, such as backache, headaches and fibromyalgia. 

Patients like Dan Schoepf, a self-employed contractor, who was first prescribed oxycodone for a severe back injury by a pain physician in Long Beach, California in 1999. As time wore on he needed ever larger doses to relieve the pain because of increasing tolerance.

“Sometimes I used up my supply of prescribed medicines too quickly and went into withdrawal, until I received my next prescription,” Schoepf says. “When I ran out, I couldn’t talk to anyone [because] I was so sick. I couldn’t live with the drugs, I couldn’t live without them and I was destroying my family.”

Then, 13 years later, Schoepf was offered physiotherapy. The pain stopped and he received addiction treatment that got him off the opioids for good.

The over-prescription of opioids for chronic, non-cancer pain outside palliative care is driving the epidemic of opioid dependency in the USA, according to Dr Debra Houry, Director of the CDC’s National Center for Injury Prevention and Control.

“When I was in medical school we didn’t know that these medications were so addictive and we were encouraged to treat pain.”

Debra Houry

“When I was in medical school we didn’t know that these medications were so addictive and we were encouraged to treat pain. Now we have better evidence that they aren’t safe for long-term prescribing,” Houry says.

The number of patients with chronic pain prescribed opioids for long periods of time has increased in other high-income countries as well, including Australia and the United Kingdom of Great Britain and Northern Ireland. But it is mainly the USA and Canada that are seeing epidemics of dependency and a large toll of overdose deaths.

Meanwhile, in much of the rest the world, there is little or no access to opioid analgesics for patients dying of cancer, even though codeine and morphine are included on WHO’s model list of essential medicines for use in palliative care. 

Health-care providers underestimate the risk of addiction of opioids and they overestimate the benefits, says Houry, a former emergency room physician.

Getting doctors to consider alternatives to opioids is a strategy Houry’s group at the CDC is taking, one of the many USA agencies tasked with fighting the opioid dependence epidemic, including the Department of Justice and the Office of the President. 

“Alternative methods other than opioids should be used first. Instead the very first thing … doctors [in this country] do is go straight to the opiates and straight to the oxy,” Schoepf says, referring to oxycodone, a commonly prescribed opioid.

In Canada, prescription opioid overdose deaths increased fivefold in the province of Ontario between 1991 and 2014 and about 2000 Canadians died from this cause in 2015 alone.

 “This enormous problem has arisen from severe and systematic medical system and practice excesses, combined with ineffective regulatory and policy actions,” says Benedikt Fischer, a senior researcher at Toronto’s Centre for Addiction and Mental Health.

In the USA, the CDC recently launched a programme that aims to improve the performance of underused prescription drug monitoring programmes. Under these programmes, state-run electronic databases are used by doctors and sometimes nurses to track the prescribing and dispensing of controlled prescription drugs to patients so that suspected abuse can be identified.

Some USA states link prescription drug monitoring programmes with patient electronic medical health records, so that doctors can more easily look up a patient’s prescription history. Others alert physicians when they see that one of their patients has been requesting prescriptions from different providers, known as “doctor shopping”.

Not all prescription drug monitoring programmes let physicians see what other prescriptions patients are receiving in real-time. Where such programmes exist, they are not used enough, Fischer adds.

Medical examiners and coroners can also use prescription drug monitoring programmes to find out which doctors may have prescribed opioids recently to a patient.

Last year, CDC released guidelines recommending evidence-based pain management techniques, such as physical therapy; and non-opioid medications, such as anti-inflammatories, as first-line treatment for chronic pain.

If prescribing opioids, it recommended that physicians ensure that patients are fully aware of the risks, start them at the lowest possible effective dose and avoid doses above a certain level. CDC is also working with medical schools and nursing colleges to ensure that students are taught safe opioid prescription and pain management.

In addition, CDC launched the Prevention for States programme in 2015 supporting 16 of the country’s 50 federal states to prevent prescription drug overdoses. It supports interventions such as incorporating CDC guidelines into health-care systems, carrying out policy re-evaluations to assess whether to use naloxone, an overdose reversal drug, and gathering data to better target overdose hotspots.

Data on the effects of these initiatives on opioid deaths are still being gathered but preliminary reports are promising, says Houry. Physicians in the State of Illinois, for example, used the prescription drug monitoring programme three times more per month compared with the previous year.

Some analyses suggest that a similar trend of prescription drug overdoses is under way in the United Kingdom of Great Britain and Northern Ireland (UK). Dr Cathy Stannard, a pain consultant in the United Kingdom, dismisses these saying that there are some prescription painkiller deaths in the country, but that adherence to guidelines prevents more overdoses and deaths. 

Deaths from the synthetic opioid painkiller, tramadol, increased between 1996, when one death was reported, and 2014 when 240 deaths were reported, according to the UK Office of National Statistics. In 2015, tramadol overdose deaths decreased to 208. 

“The National Health Service structure in the United Kingdom, where general practitioners tend to have close relationships with patients, might help restrict doctor-shopping too,” says Stannard, a consultant in Complex Pain and the Pain Transformation Programme Clinical Lead at the National Health Service Gloucestershire Clinical Commissioning Group.

“The solution lies in reforming the health-care system to incentivize doctors to talk to patients, educate patients and spend more time with them, not just to prescribe pills and perform surgeries.”

Anna Lembke

Stronger health and social systems are needed in the USA to address the prescription opioid epidemic, according to Dr Anna Lembke, whose book *Drug dealer MD *published last year, highlights the country’s epidemic of prescription opioid dependency. 

 “The solution lies in reforming the health-care system to incentivize doctors to talk to patients, educate patients and spend more time with them, not just to prescribe pills and perform surgeries,” says Lembke, an Assistant Professor of Psychiatry and an addiction specialist at Stanford University.

Many doctors do not know how to diagnose or treat addiction and are unable to help patients who become dependent on opioids. A new approach to managing pain is needed, she says: “We need to change our narratives around pain, encourage notions of resilience and understand the limits of modern medicine”. 

**Figure:**
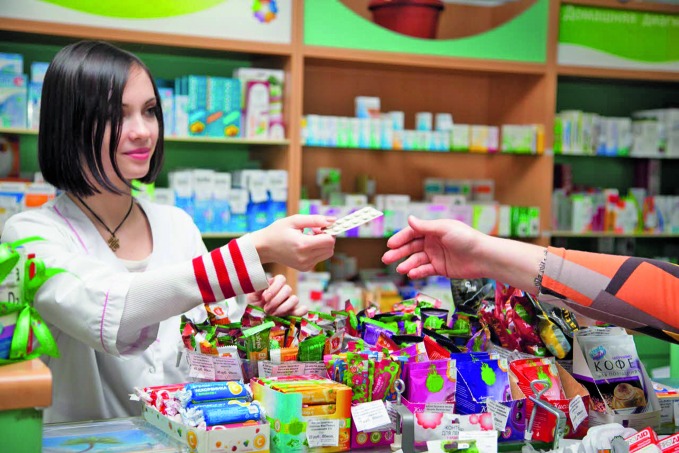
A customer receives a blister pack of pills in a pharmacy.

WHO/S. Volkov

**Figure:**
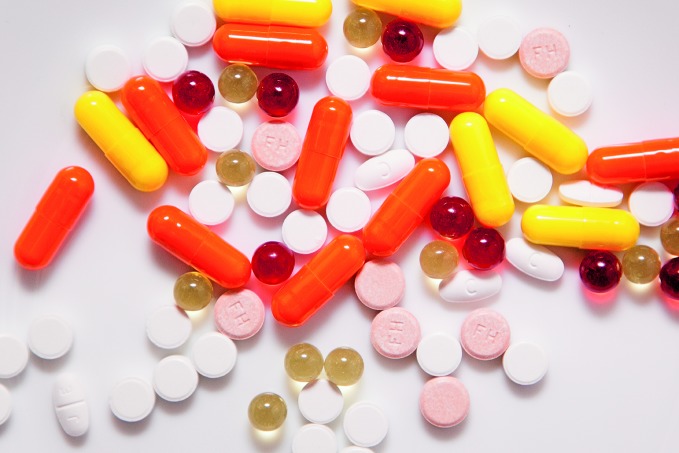
Prescription opioids are taken in the form of pills.

WHO/S. Volkov

